# Sensor-Based Human Activity and Behavior Research: Where Advanced Sensing and Recognition Technologies Meet

**DOI:** 10.3390/s23010125

**Published:** 2022-12-23

**Authors:** Hui Liu, Hugo Gamboa, Tanja Schultz

**Affiliations:** 1 Cognitive Systems Lab, University of Bremen, Enrique-Schmidt-Str. 5, 28359 Bremen, Germany; 2 LIBPhys (Laboratory for Instrumentation, Biomedical Engineering and Radiation Physics), NOVA School of Science and Technology (Campus de Caparica), 2829-516 Caparica, Portugal

## 1. Introduction

Human activity recognition (HAR) and human behavior recognition (HBR) have been playing increasingly important roles in the digital age. High-quality sensory observations applicable to recognizing users’ activities and behaviors, including electrical, magnetic, mechanical (kinetic), optical, acoustic, thermal, and chemical biosignals, are inseparable from sensors’ sophisticated design and appropriate application.

Traditional sensors suitable for HAR and HBR, including external sensors for smart homes, optical sensors such as cameras for capturing video signals, and bioelectrical, biomagnetic, and biomechanical sensors for wearable applications, have been studied and verified adequately. They continue to be researched in-depth for more effective and efficient usage, and brand new areas facilitated by sensor-based HAR/HBR are emerging, such as interactive edutainment [1], single motion duration analysis [2], time series information retrieval [3], handcrafted and high-level feature design [4,5,6], and fall detection [7]. Meanwhile, innovative sensor research for HAR or HBR is also very active in the academic community, including new sensors appropriate for HAR/HBR, new designs and applications of the above-mentioned traditional sensors, and the usage of non-traditional HAR/HBR-related sensor types, among others.

This Special Issue aims to provide researchers in related fields with a platform to demonstrate their unique insights and late-breaking achievements.

## 2. Overview of the Contributions

Ten high-quality representative articles, including eight research papers and two surveys, have undergone a rigorous selection and review process to be published in this Special Issue. Although it cannot be said that they cover all aspects of the topic “sensors for human activity recognition,” they reflect the latest developments in the field in a high-profile manner.

We follow the sequence of tasks indicated by the state-of-the-art HAR research pipeline [8,9] to organize and introduce these contributions. Therefore, it should be emphasized that the order in which the articles appear does not correlate with their academic value.

### 2.1. Hardware Preparation: Sensing Technologies and Camera Calibration Technologies

As the hardware cornerstone of all relevant research areas, sensing technology is a topic that cannot be jumped. We are delighted to have such an opening article in this Special Issue [10], which presented a thorough, in-depth survey on the state-of-the-art sensing modalities in HAR tasks to supply a solid understanding of the variant sensing principles for younger researchers of the community. The HAR-related sensing modalities are reasonably categorized into five classes: mechanical kinematic sensing, field-based sensing, wave-based sensing, physiological sensing, and hybrid/others, with the strengths and weaknesses of each modality across the categorization compared and discussed to provide newcomers with a better overview and references.

Equipped with advanced knowledge of sensing technology, researchers step into the door of sensor-based HAR to select appropriate high-quality sensors for their research scenarios. Afterward, preparatory work is required to acquire continuous, high-quality biosignals. For example, the calibration task is essential for video camera-based HAR, where high-precision distortion calibration is a prerequisite for perfect activity recognition with external sensing. Conventional approaches sometimes need hundreds or thousands of images to optimize the camera model. Jin et al. put forward an innovative point-to-point distortion calibration procedure that requires only dozens of images to obtain a dense distortion rectification map, contributing to a 28.5% enhancement to the reprojection error over the polynomial distortion model [11]. It is worthy of academic focus that although the authors emphasized the application of the new method in HAR, we find that the applicability is not limited to HAR and deserves a more extensive scope of attention.

### 2.2. Signal Processing: Traditional Feature Extraction versus Deep Neural Representations

After the aspects of devices have been adequately studied, sensor signals will be acquired, archived, and analyzed. Digital signal processing (DSP) techniques are widely used in the subsequent stage, among which feature extraction serves as the bridge that connects the raw data with machine learning. Traditional machine learning for HAR generally employs conventional features in statistical, temporal, and frequency domains to execute experiments such as combination, selection, stacking, and dimensionality reduction. In contrast, deep learning requires deep neural representations. It is a very arresting academic work to compare the two through a new and rational approach [12]. The work analyzes both approaches in multiple domains utilizing homogenized public datasets, verifying that even though deep learning initially outperforms handcrafted features, the situation is reversed as the distance from the training distribution increases, which supports the hypothesis that handcrafted features may generalize better across specific domains.

### 2.3. Recognition and Localization on Human Activities or Behaviors: Deep Learning versus No Training

The vast majority of sensor-based HAR tasks rely on machine learning. Despite the irreplaceable advantages of traditional feature-based machine learning suggested in Section 2.2, deep learning is increasingly demonstrating its powerful adaptive capabilities. Besides [12], this Special Issue contains three more articles on deep learning [13,14,15], offering us multiple dimensions of thinking:

- The training of HAR models requires a large amount of annotated data corpus. Most current models are not robust when facing anonymized data from new users; meanwhile, capturing each new subject’s data is usually not possible. Yang et al. described semi-supervised adversarial learning using the long-short term memory (LSTM) approach for HAR [13], which trains labeled and anonymous data by adapting the semi-supervised learning paradigms on which adversarial learning capitalizes to enhance the learning capabilities handling errors.
- The device-free, privacy-protected, and light-insensitive characteristics have pushed WIFI-based HAR technology into the limelight. Evolving machine learning techniques have significantly improved sensing accuracy with existing methods. To improve the performance of the challenging multi-location recognition, researchers in [14] proposed an amplitude- and phase-enhanced deep complex network (AP-DCN) for multi-location HAR to exploit the amplitude and phase information synchronously and thus retrieve richer information from limited samples. A perception method based on a deep complex network-transfer learning (DCN-TL) structure was practiced to effectively achieve knowledge sharing among multiple locations, aiming to address the imbalance in sample numbers.
- Sensor-based indoor localization is also relevant to this Special Issue’s scope by using acceleration signals to represent human behavior to be useful additional information on GPS signals for modeling and learning. Sensor-based indoor localization is also relevant to this Special Issue by using acceleration signals to represent human behavior to be useful additional information on GPS signals for modeling and learning. Study [15] exhibited a pedestrian dead reckoning-based indoor localization system on a smartphone, where accelerometer and GPS data were used as input and labels to estimate moving speed through deep learning. A distance error of approximately 3 to 5 m in the experiments within a 240 m-long horseshoe-shaped building is a welcome level of accuracy.

Model training for machine learning, or, furthermore, deep learning, is not the sole path to reaching human activity or behavior recognition. We have a research piece that seeks to achieve behavioral recognition without training [16]. The convenience store is a daily business form worldwide; for Japanese society, its transliteration abbreviation “Kombini” goes beyond shopping, representing a unique cultural and emotional sustenance. Recognizing customer behaviors in monitoring videos can supply analytical material for business management in smart retail solutions. Therefore, the scientists from Japan put the analysis of human behavior in the convenience store scene, which is an appropriate and attractive setting. Unlike previous approaches based on model training, customer behavior in this research is combined with primitives to achieve flexibility, where a primitive is a unit describing an object’s motion or multiple objects’ relationships.

### 2.4. Evaluating the Experimental Results: Also an Essential Research Topic

Besides the machine learning task for HAR, what research can be further executed before practical applications? In [17], the authors proposed explainable methods to understand the performance of mobile HAR mobile systems according to the chosen validation strategies. A novel approach, SHAP (Shapley additive explanations), was used to discover potential bias problems of accuracy overestimation based on the inappropriate choice of validation methodology. We believe this study is academically significant and of guiding value for practice.

### 2.5. More Referential Topics Linked to Body Sensor Networks and Human Physiological Signals

As an academic expansion, we have also selected two articles about wearable sensors combined with human physiological signal applications to benefit our readers. They provide sensor-based human activity/behavior researchers with references and inspirations for the device and experimental design.

Authors of [18] successfully modeled subjects’ psychological stress in different states through electrocardiogram (ECG) signals during a virtual reality high-altitude experiment. Participants wore in-house-designed smart T-shirts with embedded multiple sensors to complete different tasks. A deep, gated recurrent unit (GRU) neural network was developed to capture the mapping between subjects’ ECG and stress represented by heart rate variability (HRV) features.

The ankle joint, one of the body’s most important joints for maintaining the ability to walk, can be damaged due to stroke or osteoarthritis, which will cause gait disturbances. Ankle-foot orthoses have been widely applied to help patients regain their natural gait. Article [19] reviewed the development of ankle-foot orthoses and prospected combining ankle-foot orthoses with rehabilitation techniques, such as myoelectric stimulation, to reduce the energy expenditure of patients in walking.

## 3. Conclusions, Outlook, and Acknowledgments

Having attracted many contributions from outstanding world scientists, this Special Issue has become thriving and full of academic tensions. Given the enthusiasm of the submissions, the second volume of this Special Issue has been launched, and we look forward to more scholars publishing their admiring contributions.

We pay special tribute and appreciation to all 51 authors of the articles, as well as all professional and diligent reviewers. 
